# GSDMD/Drp1 signaling pathway mediates hippocampal synaptic damage and neural oscillation abnormalities in a mouse model of sepsis-associated encephalopathy

**DOI:** 10.1186/s12974-024-03084-w

**Published:** 2024-04-16

**Authors:** Qun Fu, Yi-Bao Zhang, Chang-Xi Shi, Ming Jiang, Kai Lu, Zi-Hui Fu, Jia-Ping Ruan, Jing Wu, Xiao-Ping Gu

**Affiliations:** 1 Department of Anesthesiology, Nanjing Drum Tower Hospital, Affiliated Hospital of Medical School, Nanjing University, 321 Zhongshan Road, Nanjing, 210008 China; 2 Department of Anesthesiology, Henan Provincial Chest Hospital, Zhengzhou University, 1 Weiwu Road, Zhengzhou, 450000 China; 3 Jiangsu Key Laboratory of Molecular Medicine, Medical School of Nanjing University, Nanjing, 210008 China

**Keywords:** Sepsis-associated encephalopathy, Hippocampus, GSDMD, Dynamin-related protein 1, Cognitive impairment

## Abstract

**Background:**

Gasdermin D (GSDMD)-mediated pyroptotic cell death is implicated in the pathogenesis of cognitive deficits in sepsis-associated encephalopathy (SAE), yet the underlying mechanisms remain largely unclear. Dynamin-related protein 1 (Drp1) facilitates mitochondrial fission and ensures quality control to maintain cellular homeostasis during infection. This study aimed to investigate the potential role of the GSDMD/Drp1 signaling pathway in cognitive impairments in a mouse model of SAE.

**Methods:**

C57BL/6 male mice were subjected to cecal ligation and puncture (CLP) to establish an animal model of SAE. In the interventional study, mice were treated with the GSDMD inhibitor necrosulfonamide (NSA) or the Drp1 inhibitor mitochondrial division inhibitor-1 (Mdivi-1). Surviving mice underwent behavioral tests, and hippocampal tissues were harvested for histological analysis and biochemical assays at corresponding time points. Haematoxylin-eosin staining and TUNEL assays were used to evaluate neuronal damage. Golgi staining was used to detect synaptic dendritic spine density. Additionally, transmission electron microscopy was performed to assess mitochondrial and synaptic morphology in the hippocampus. Local field potential recordings were conducted to detect network oscillations in the hippocampus.

**Results:**

CLP induced the activation of GSDMD, an upregulation of Drp1, leading to associated mitochondrial impairment, neuroinflammation, as well as neuronal and synaptic damage. Consequently, these effects resulted in a reduction in neural oscillations in the hippocampus and significant learning and memory deficits in the mice. Notably, treatment with NSA or Mdivi-1 effectively prevented these GSDMD-mediated abnormalities.

**Conclusions:**

Our data indicate that the GSDMD/Drp1 signaling pathway is involved in cognitive deficits in a mouse model of SAE. Inhibiting GSDMD or Drp1 emerges as a potential therapeutic strategy to alleviate the observed synaptic damages and network oscillations abnormalities in the hippocampus of SAE mice.

**Supplementary Information:**

The online version contains supplementary material available at 10.1186/s12974-024-03084-w.

## Background

Sepsis-associated encephalopathy (SAE) is characterized by diffuse cerebral dysfunction and irreversible cognitive impairment, resulting from systemic inflammation, even in the absence of direct central nervous system infection. It manifests in approximately 53% of patients with sepsis [1]. Survivors of sepsis could develop various cognitive deficits, including memory, attention, executive function, and speed of information processing [2]. Notably, these mental states are closely associated with the hippocampus [3, 4]. The pathogenesis of SAE is multifarious, involving factors such as blood‒brain barrier (BBB) dysfunction, neuroinflammation [5], neurotransmitter dysfunction, alterations in synaptic plasticity, and mitochondrial dysfunction [5, 6]. Despite these associations, the molecular and cellular mechanisms underlying SAE are poorly understood [7], and effective therapies for SAE are currently lacking.

The gasdermin D (GSDMD) protein acts as a crucial mediator of pyroptosis, causing cell membrane disruption upon cleavage by caspase-1/-11 [8, 9]. This process triggers robust inflammation by releasing proinflammatory cytokines, making GSDMD a promising therapeutic target. Our previous study demonstrated that sepsis-triggered activation of the canonical nucleotide-binding domain-like receptor protein 3 (NLRP3)-caspase-1 inflammasome cleaves GSDMD in the hippocampus, initiating neuronal pyroptosis and neuroinflammation, leading to hippocampus-dependent memory impairments [10]. Additionally, pharmacological inhibition of the NLRP3-caspase-1 inflammasome has demonstrated a reduction in the expression of both GSDMD and its cleavage form, GSDMD-N, effectively mitigating pyroptosis in the mouse brain following sepsis [10, 11]. Furthermore, GSDMD knockout mice exhibited improved behavioral outcomes after sepsis [12].

A previous study has demonstrated the potential involvement of GSDMD-N in mediating mitochondrial dysfunction [13]. Moreover, it has been proposed that mitochondrial reactive oxygen species (ROS) could guide GSDMD-N to the mitochondria, leading to a switch in cell death modality in leucine-rich repeat kinase 2 (Lrrk2) macrophages. This process is proposed to require the participation of mitochondrial dynamin-related protein 1 (Drp1) [14]. Drp1 is a cytosolic GTPase that has been suggested to couple GTP hydrolysis to membrane constriction and fission [15]. Recent evidence has demonstrated the translocation of Drp1 to mitochondria, where it mediates mitochondrial fission [16]. Furthermore, Drp1 may interact with Lrrk2 to drive Drp1 into mitochondria, ultimately leading to aberrant mitochondrial fission [17].

Mitochondria, vital for various cellular functions like ATP generation, ROS production, and ion homeostasis, are implicated in the pathophysiology of sepsis-triggered neuroinflammation and programmed cell death [18]. Recent findings suggest that mitochondrial dysfunction can trigger apoptosis, oxidative phosphorylation, and neuroinflammation, potentially leading to a positive feedback loop of mitochondrial dysfunction [19, 20]. Alterations and damage to mitochondrial dynamics are linked to several neurodegenerative diseases [21, 22]. In particular, Drp1 may control mitochondrial function and the balance of mitochondrial fission/fusion, which is necessary for brain function and energy supply [23]. Moreover, mitochondrial amyloid-β (Aβ) has been linked to Drp1 activation, exacerbating mitochondrial dysfunction, and triggering NLRP3 inflammasome activation, leading to an increase in cleaved GSDMD [24]. Considering the high energy demands of neural oscillation, which are met by mitochondria, targeting Drp1 may emerge as a therapeutic strategy to prevent abnormal mitochondrion-related neurodegenerative diseases like Alzheimer’s disease (AD) and Parkinson’s disease (PD) [25].

Necrosulfonamide (NSA), a specific GSDMD inhibitor, has demonstrated neuroprotective effects in various neurodegenerative and neuropsychiatric diseases by mitigating pyroptosis and neuroinflammation [26]. NSA has also shown efficacy in reducing neuronal death and neuroinflammation in a mouse intracranial hemorrhage model [27]. Mitochondrial division inhibitor-1 (Mdivi-1) is a Drp1-specific inhibitor. Inhibition of Drp1 with Mdivi-1 has been reported to ameliorate abnormal mitochondrial dynamics [28] and mitochondrial dysfunction induced by neuroinflammation [29]. However, the protective roles of the NSA and Mdivi-1 in SAE are still underestimated.

Currently, no effective strategies exist to prevent or slow the progression of SAE. Hence, this study aimed to explore the neuronal mechanisms and signaling pathways underlying cognitive impairments in a mouse model of SAE. Based on existing evidence, we hypothesized that the administration of NSA and Mdivi-1 could mitigate cognitive deficits induced by the GSDMD/Drp1 signaling pathway in septic mice.

## Materials and methods

### Animals

One hundred and eighty C57BL/6 male mice, aged 10–12 weeks, were purchased from the Animal Center of the Nanjing Drum Tower Hospital, Affiliated Hospital of Medical School, Nanjing University, China. All animal procedures were performed according to the Guidelines for the Care and Use of Laboratory Animals from the National Institutes of Health and with approval of the Committee of the Nanjing Drum Tower Hospital, Affiliated Hospital of Medical School, Nanjing University. Mice were housed in groups of four individuals per cage with a 12 h light/dark cycle (lights on at 8 a.m. and off at 8 p.m.) at a room temperature of 22 ± 1 °C and a humidity of 50–60% and with *ad libitum* access to standard food and water. All mice had acclimated to the environment for at least one week prior to experiments.

### Cecal ligation and puncture (CLP) model

CLP was performed after anesthesia using 2% sodium pentobarbital (40 mg/kg, Sigma, St. Louis, MO, USA) intraperitoneally (*i.p.*) as described in our previous studies [10]. Briefly, after intraperitoneal anesthesia, a midline incision (1.5 cm) was made in the lower abdomen. The caecum was carefully isolated under aseptic conditions and ligated using 4.0 silk suture below the ileocecal junction, approximately 1 cm from the distal end. Subsequently, the caecum was perforated with a sterile 22-gauge needle, and fecal contents were extruded through the puncture pore by gently squeezing the caecum. Finally, the intestinal tract was returned to the peritoneal cavity, and the abdomen was sutured with 4.0 silk sutures. In sham operative mice, the caecum was exposed in the same way as in CLP, but it was neither ligated nor punctured. Mice were immediately revived with a subcutaneous injection of saline solution (30 ml/kg) after surgery and returned to their cages.

### Experimental design

**Fig. 1:**
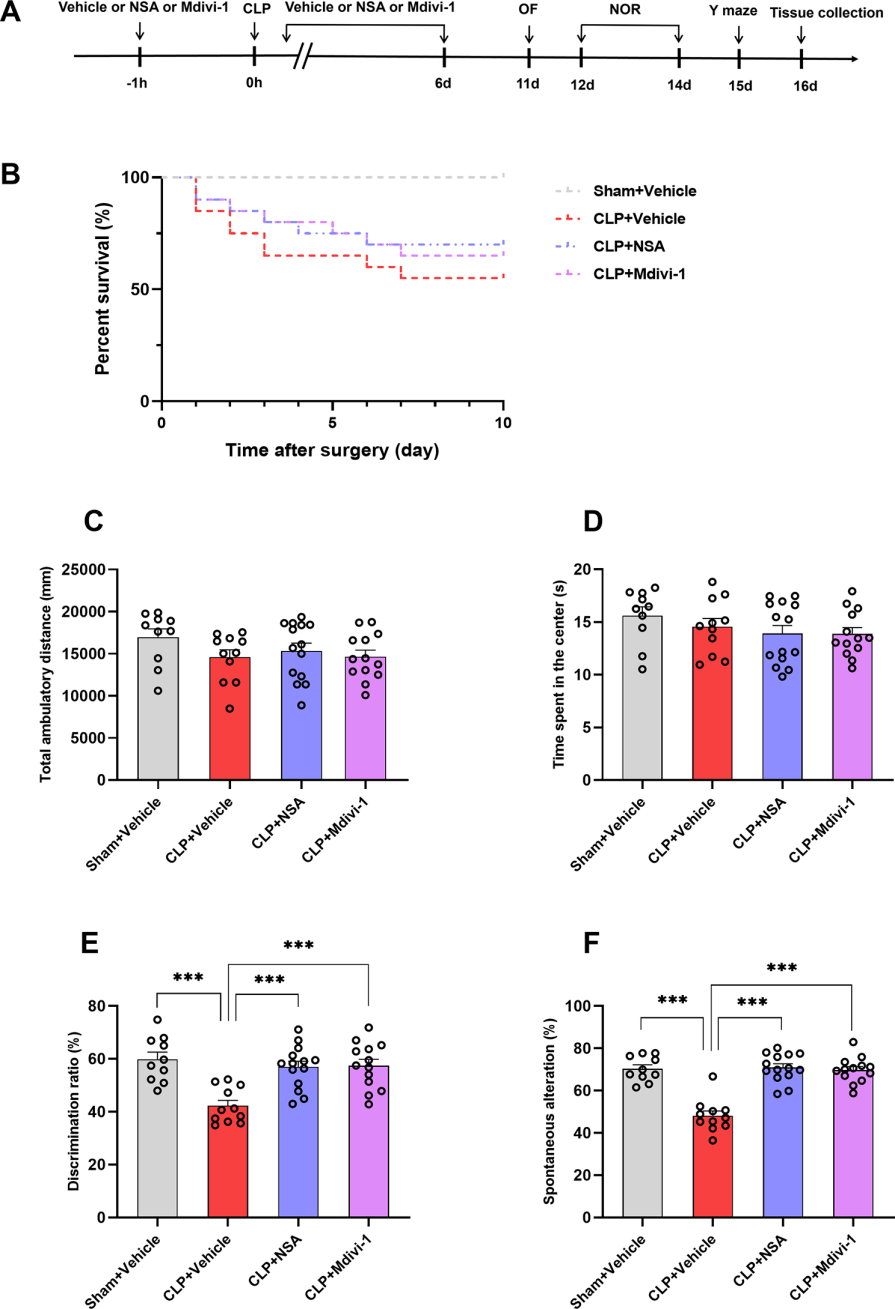
NSA or Mdivi-1 improved learning and memory in the septic mice after CLP. **A** Schematic timeline of the experimental procedure. **B** Survival rate within 10 days (*n* = 10 mice/sham subgroup; *n* = 20 mice/CLP subgroup). **C** Total ambulatory distance in open field tests. **D** Time spent in the center in open field tests. **E** Discrimination ratio in the novel object recognition tests. **F** Spontaneous alterations in Y maze tests. Data are presented as the mean ± SEM (*n* = 10–14 mice/group). ^***^P < 0.001 versus the indicated groups

In experiment 1, mice were randomly assigned to the following groups: sham + vehicle (*n* = 10), sham + NSA (*n* = 10), sham + Mdivi-1 (*n* = 10), CLP + vehicle (*n* = 20), CLP + NSA (*n* = 20), and CLP + Mdivi-1 (*n* = 20). Surviving mice underwent behavioral tests and hippocampal tissues were collected for histological analysis. The experimental protocol is presented in Fig. 1A.

In experiment 2, mice were randomly assigned to sham + vehicle (*n* = 8), CLP + vehicle (*n* = 16), CLP + NSA (*n* = 16), and CLP + Mdivi-1 (*n* = 16). Surviving mice underwent biochemical assays on postoperative day 7. In addition, 6 mice were used for co-immunoprecipitation.

In experiment 3, mice were randomly assigned to sham + vehicle (*n* = 4), CLP + vehicle (*n* = 8), CLP + NSA (*n* = 8), and CLP + Mdivi-1 (*n* = 8). Surviving mice underwent in vivo electrophysiology. The experimental protocol is presented in Fig. 7A. The number of animals used in each experiment is based on our previous studies [7, 10] and relevant researches [11, 30, 31] in the field.

Mice in the pharmacological intervention groups were administered either vehicle 1% dimethyl sulphoxide (DMSO), NSA (5 mg/kg, no. HY-100,573; MedChemExpress, Monmouth Junction, NJ, USA) [27] or Mdivi-1 (10 mg/kg, no. T1907; Topscience Co., Ltd., Shanghai, China) by intraperitoneal injection 1 h before surgery and then once daily for the subsequent 6 consecutive days [32]. Both compounds are capable of crossing the BBB [32, 33].

### Open field test

The open field (OF) test was carried out in a white opaque plastic chamber (40 × 40 × 40 cm) to assess the locomotor and exploratory activities of mice. Each mouse was gently positioned at the center of the arena for 5 min, and its activity was automatically recorded using a video tracking system (XR-XZ301, Shanghai XinRuan Information Technology Co., Ltd., Shanghai, China). All tests were conducted between 8 a.m. and 5 p.m. To eliminate any olfactory cues, the experimental apparatus was cleaned with a solution of 75% ethanol after each trial.

### Novel object recognition

The novel object recognition (NOR) test was performed in a square plastic apparatus (40 × 40 × 40 cm) to evaluate learning and memory ability of mice. Before the test, animals were accustomed to two identical objects in the open box and allowed to explore for 10 min. Subsequently, one of the two objects was replaced by a novel subject with a distinct shape and color. The time spent exploring the familiar and novel subjects was recorded automatically. The discrimination ratio was calculated as the time spent exploring the novel object/ (time spent exploring the novel object + time spent exploring the old object). The apparatus was thoroughly cleaned with 75% ethanol between each test.

### Y maze

The Y maze, consisting of three arms at 120° angles labelled A, B, and C, was used to evaluate the spatial working memory of mice. Each animal was placed in the center of the equipment and allowed to explore freely for 8 min throughout the three different arms of the Y maze. The sequence and total number of arms entered were recorded. When the hind paws of the animal had been completely placed in the arm, arm entry was complete. For instance, a sequence of entries to the three arms ABC, ACBABACABA, would generate four “successful” alternations, ACB, CBA, BAC, and CAB. The score of alternation was calculated as the number of triads containing entries into all three arms divided by the maximum possible alternations (the total number of arms entered minus 2) × 100. Re-entry into the same arm was not counted for the analysis. The experimental equipment was thoroughly cleaned with 75% alcohol after each test.

### Haematoxylin and eosin (HE) staining

Mice were deeply anaesthetized using 2% sodium pentobarbital (60 mg/kg, *i.p.*; Sigma, St. Louis, MO, USA). The whole brain was removed and fixed in 4% paraformaldehyde and embedded in paraffin for HE staining using a Hematoxylin-Eosin Stain kit (Nanjing Jiancheng Bioengineering Institue, Nanjing, China). The HE staining procedure included dyeing with haematoxylin for 5 min, decolorization with a 75% hydrochloric acid alcohol solution for 30 s; eosin staining for 5 min, and final decolorization with 90% ethanol for 35 s. Hippocampal neuronal damage was assessed with a standard semiquantitative scale [10, 34]. In brief, 0 indicates no lesions in the hippocampal CA1 region, 1 indicates scattered damaged neurons in the hippocampal CA1 region, 2 indicates moderate numbers of damaged neurons in the hippocampal CA1 region (< 50% neurons affected), 3 indicates severe damage in the hippocampal CA1 region (> 50% of cells damaged), and grade 4 indicates extensive neuronal damage in the hippocampal regions. Evaluation was evaluated by an investigator blinded to each group.

### Immunofluorescence staining

Coronal brain sections with a thickness of 10 μm were prepared and placed onto glass slides. Slices were blocked with 1% bovine serum albumin (BSA) for 1 h at room temperature. Subsequently, sections were incubated overnight at 4 °C with rabbit anti-ionized calcium-binding adaptor molecule-1 (Iba-1; 1:200, CY7217, Abways, Shanghai, China); rabbit anti-glial fibrillary acidic protein (GFAP; 1:200, BA0056, Boster, Pleasanton, CA, USA); rabbit anti-Drp1 (1:500, BP51203, Abbkine Scientific Co., Ltd., Wuhan, China); and rabbit anti-GSDMD (1:100, A22602, ABclonal Technology Co., Ltd., Wuhan, China) overnight at 4 °C, followed by a 1 h incubation with donkey anti-rabbit IgG-FITC (1:500, Abbkine Scientific Co., Ltd., Wuhan, China) or goat anti-rabbit IgG-Cy3 (1:600, Abbkine Scientific Co., Ltd., Wuhan, China) at room temperature. For mounting and counterstaining, sections were incubated with DAPI (1:1000, Beyotime Biotechnology, Shanghai, China). Fluorescence images were captured using a confocal microscope (Leica, TCS SP2, Germany), and ImageJ software (National Institutes of Health, Bethesda, MD, USA) was utilized to detect the mean value of the immunofluorescence intensity in each section.

### TUNEL staining

Neuronal cell death in the hippocampus was assessed using terminal deoxynucleotidyl transferase-mediated dUTP nick-end labeling (TUNEL) assays with One-step TUNEL Apoptosis Assay Kit (red TRITC labeled fluorescence) (KGA7063, Jiangsu KeyGen Biotech Co., Ltd., Nanjing, China) [35]. The sections were first incubated with rabbit anti-NeuN (1:400, 26975-1-AP, Proteintech, Wuhan, China), followed by TUNEL reagents according to the manufacturer’s instructions. The counts of TUNEL-positive neuron in the hippocampus CA1 region were evaluated by an investigator blinded to the intervention conditions.

### Transmission electron microscopy

After anesthesia using 2% sodium pentobarbital, mice were perfused intracardially with phosphate-buffered saline (PBS). Hippocampi were removed and immediately put in petri dishes with transmission electron microscopy (TEM) fixative, cut into 1 mm^3^ tissue blocks, and then preserved at 4 °C. The tissues were postfixed with 1% osmium tetroxide in 0.1 M PBS (pH 7.4) for 2 h at room temperature, followed by rinsing in 0.1 M PBS 3 times for 15 min each. Dehydration was achieved through a graded ethanol series, followed by resin embedding, and cutting into 60–80 nm thin sections using an ultramicrotome. The sections were placed on 150-mesh copper grids with formvar film. Staining involved 2% uranium acetate saturated alcohol solution for 8 min, followed by rinsing in 70% ethanol and ultrapure water. Lead citrate (2.6%) was used for 8 min to avoid CO_2_ staining, with subsequent rinsing in ultrapure water 3 times. After drying with filter paper, the cuprum grids were placed on a grid board and dried overnight at room temperature. The cuprum grids were observed under TEM, and images were taken.

### Golgi-Cox staining

On Day 16 post-surgery, mouse brains were subjected to Golgi-Cox staining using a Golgi Stain Kit (no. GP1152, Servicebio Technology Co., Ltd., Wuhan, China). In brief, following deep anesthesia with 2% sodium pentobarbital, the brains were promptly extracted and rinsed in double-distilled water. They were immersed in impregnation solutions A and B and placed in a dark, undergoing a 14-day treatment shielded from light in a dark, well-ventilated room at room temperature. Following this, the staining solution was replaced with solution C and stored for an additional 7 days. Brain tissue was sectioned into 100 microns using an oscillating microtome and mounted on gelatine slides for staining. After undergoing alcohol dehydration, the tissue sections were subsequently cleared in xylene and coverslipped. All sections were then observed using an optical light microscope (Olympus Microscope System BX51, Olympus Corporation, Tokyo, Japan), scanned using a digital slice scanner, and subsequently analyzed using Fiji software.

### Co-immunoprecipitation (Co-IP) and western blotting analysis

The obtained samples were homogenized in ice-cold lysis buffer supplemented with a protease inhibitor cocktail, followed by centrifugation at 12,000 × g for 10 min at 4 °C. The supernatant was collected, and its protein concentration was determined using the Bradford assay.

For Co-IP assay, the supernatant was subjected to anti-GSDMD or IgG control or anti-Drp1 antibody immunoprecipitation for 2 h followed by overnight incubation with protein A/G-agarose beads following the manufacturer’s instruction. The beads were washed for five times with the lysis buffer, and then boiled with the SDS loading buffer for 10 min.

For western blotting, twenty micrograms of protein were separated via SDS‒PAGE and transferred to nitrocellulose membranes. After blocking with 5% nonfat dry milk for 1 h at room temperature, the membranes were subsequently exposed to primary antibodies, including rabbit anti-GSDMD (1:1000, Ab219800, Abcam, Cambridge, UK); rabbit anti-cleaved GSDMD (1:1000, #10137S, Cell Signaling Technology, Danvers, MA, USA); rabbit anti-Drp1 (1:1000, ABP51203, Abbkine Scientific Co., Ltd., Wuhan, China); rabbit anti-interleukin-1β (IL-1β; 1:1000, GB11113, Servicebio Technology Co., Ltd., Wuhan, China); rabbit anti-IL-18 (1:1000, GB115632, Servicebio Technology Co., Ltd., Wuhan, China); rabbit anti-tumor necrosis factor-α (TNF-α; 1:1000, GB11188, Servicebio Technology Co., Ltd., Wuhan, China), and rabbit anti-β-actin (1:1000, GB15003-100, Servicebio Technology Co., Ltd., Wuhan, China). After washing in TBST three times, the membranes were incubated with goat anti-rabbit IgG-horseradish peroxidase-conjugated secondary antibodies (1:3000, Servicebio Technology Co., Ltd., Wuhan, China). The protein bands were detected via enhanced chemiluminescence and quantified utilizing ImageJ software (National Institutes of Health, Bethesda, MD, USA).

### ROS detection

Hippocampal tissues were harvested and homogenized in ice-cold lysis buffer supplemented with a protease inhibitor cocktail. Cellular ROS levels were detected using a ROS assay kit (no. S0033S, Beyotime Biotechnology, Shanghai, China) utilizing an oxidation-sensitive fluorescent probe (DCFH-DA), and measured in a spectrofluorometer (excitation 490 nm, emission 520 nm), following established protocols [7]. All measurements were performed in accordance with the manufacturer’s instructions.

### In vivo electrophysiology

Mice were anaesthetized using 2% sodium pentobarbital (40 mg/kg, *i.p.*; Sigma, St. Louis, MO, USA) while maintaining their body temperature with an electric blanket. To fully expose the skull, the muscle, fascia tissue, and periosteum were removed. After balancing the skull bone, three dimensional coordinates of the CA1 region (DP, -2.0 mm; ML, ± 1.5 mm; DV, -1.5 mm) were obtained from the mouse brain atlas. A 3 × 3 mm window was drilled on the skull above the CA1 region, and the cortex was fully exposed. A 4 × 2 electrode array with 8 microwires was used. The electrode was slowly lowered through the window to the predetermined position. After closing the window with paraffin, the electrode was fixed on the skull surface with dental cement. Raw data were recorded in NOR on Day 12 after CLP. All data analyses were performed using NeuroExplorer 5 (Plexon Inc., Dallas, TX). The signals were transmitted into a digital preamplifier and digitized at a sampling rate of 40 kHz. To prevent interference, all other electronic devices were turned off during the recording session. Data processing was performed on a specific workstation running professional electrophysiological analysis software. For the local field potential (LFP) analysis, wide band recordings were downsampled at 1250 Hz. The bands were filtered as follows: delta (0.5–3 Hz), theta (4–7 Hz), alpha (8–13 Hz), beta (14–29 Hz), and gamma (30–80 Hz) oscillations.

### Statistical analysis

Statistical analyses were performed using GraphPad Prism 8.0 (GraphPad Software, Inc.). Data are expressed as the mean ± S.E.M. Normal distribution of the data was assessed using the Kolmogorov‒Smirnov test. Survival rate was assessed by the Kaplan‒Meier method and compared among groups using the log-rank test. Differences among groups were analyzed using one-way analysis of variance (ANOVA) for normally distributed data, followed by Tukey’s multiple comparisons post hoc test, or for non-normally distributed data, followed by Fisher’s LSD test. A *p* value < 0.05 was considered statistically significant. If significant, overall effects were reported in each graph.

## Results

### Inhibiting GSDMD or Drp1 ameliorated hippocampus-dependent cognitive deficits in septic mice

In this study, mouse mortality increased for 10 days after CLP. The survival rate with intraperitoneal NSA (70%) or Mdivi-1 (65%) was slightly increased compared to the CLP + vehicle group (55%). However, there was no significant difference among the CLP subgroups (Fig. 1B). To determine the impact of CLP challenge and NSA or Mdivi-1 treatment on cognitive function, we conducted a battery of behavioral tasks as described in our previous research [10]. Locomotor activity and exploratory behavior were evaluated by the open field test on Day 11 after surgery. There was no significant difference in the total ambulatory distance [*F* (3, 44) = 1.333, *P* = 0.2760, Fig. 1C] and time spent in the center of the arena [*F* (3, 44) = 1.081, *P* = 0.3671, Fig. 1D] among the groups. The discrimination ratio was significantly decreased in the CLP + vehicle group in the NOR test, and this was reversed by administration of NSA or Mdivi-1 [*F* (3, 44) = 10.77, *P* < 0.0001, Fig. 1E]. On Day 15 after surgery, mice were evaluated by the spontaneous alternation of the Y-maze paradigm, which assesses spatial working memory. The mice in the CLP + vehicle group displayed less spontaneous alteration than the mice in the sham + vehicle group, and this effect was reversed by NSA or Mdivi-1 treatment [*F* (3, 44) = 32.24, *P* < 0.0001, Fig. 1F].

As NSA or Mdivi-1 did not influence the behavioral analysis of mice after sham surgery, we omitted the control group of sham + NSA and sham + Mdivi-1 in a follow-up experiment. There were no significant differences in the total ambulatory distance [*F* (2, 27) = 0.0777, *P* = 0.9254, Supplementary Fig. 1A], time spent in the center of the arena [*F* (2, 27) = 1.972, *P* = 0.1587, Supplementary Fig. 1B], discrimination ratio [*F* (2, 27) = 0.3532, *P* = 0.7057, Supplementary Fig. 1C], and spontaneous alternation [*F* (2, 27) = 0.1084, *P* = 0.8976, Supplementary Fig. 1D] among the sham subgroups.

### Inhibiting GSDMD or Drp1 suppressed GSDMD/Drp1 pathway activation in the hippocampus of SAE mice

**Fig. 2:**
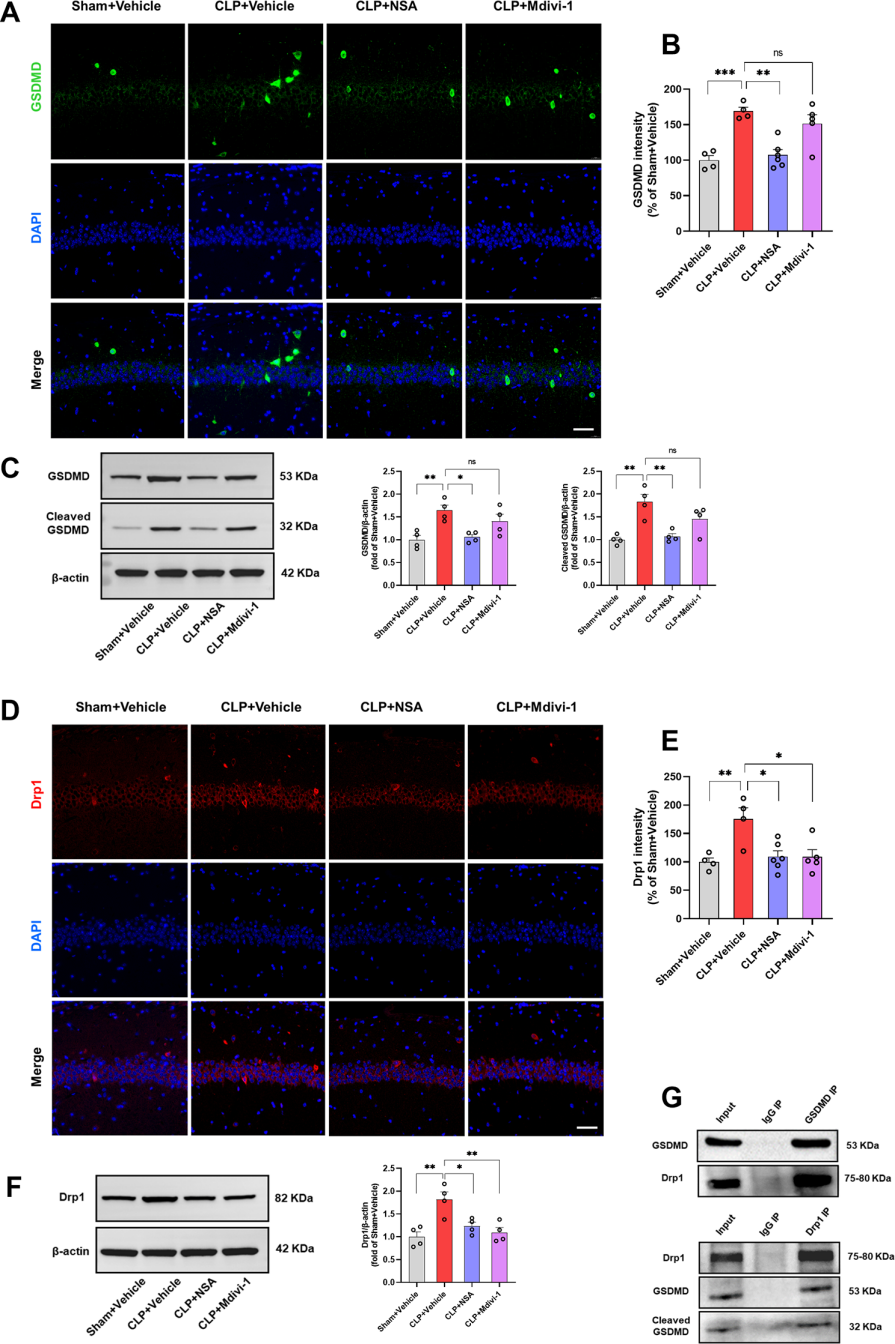
NSA or Mdivi-1 inhibited the activation of GSDMD/Drp1 signal pathway in the hippocampus of SAE mice. **A** Representative images of GSDMD (green) in the hippocampal CA1 region. **B** Quantification of GSDMD fluorescence in the hippocampal CA1 region. Data are presented as the mean ± SEM (*n* = 4–6 mice/group). DAPI staining is shown in blue. Scale bar = 50 μm. **C** Representative western blotting and quantitative analysis of the protein levels of GSDMD and cleaved GSDMD (GSDMD-N) in the hippocampus. Data are presented as the mean ± SEM (*n* = 4 mice/group). **D** Representative images of Drp1 (red) in the hippocampal CA1 region. **E** Quantification of Drp1 fluorescence in the hippocampal CA1 region. Data are presented as the mean ± SEM (*n* = 4–6 mice/group). **F** Representative western blotting and quantitative analysis of the protein levels of Drp1 in the hippocampus. Data are presented as the mean ± SEM (*n* = 4 mice/group). ^*^P < 0.05, ^**^P < 0.01, ^***^P < 0.001 versus the indicated groups. **G** Immunoprecipitation of GSDMD and Drp1 from mouse hippocampus (*n* = 6). Hippocampal lysates were subjected to immunoprecipitation using either an anti-GSDMD antibody, or an anti-Drp1 antibody or IgG control, followed by western blotting with anti-Drp1 and anti-GSDMD antibodies

Our previous study demonstrated that sepsis-triggered canonical NLRP3 inflammasome activation cleaved GSDMD in the hippocampus, inducing neuronal pyroptosis and neuroinflammation, causing hippocampus-dependent memory impairments [10]. This study investigates the involvement of GSDMD in mitochondrial damage during SAE, with a specific focus on its interaction with Drp1, a crucial regulator of mitochondrial fission [36], linked to neuroinflammation and critical roles in cellular homeostasis, synapse formation and postoperative cognitive dysfunction (POCD) [32]. Immunostaining using an antibody against GSDMD in the mouse hippocampus revealed that CLP induced an increase in the mean intensity of GSDMD, a change reversed by NSA but not by Mdivi-1 in the hippocampal CA1 region [*F* (3, 15) = 13.13, *P* = 0.0002, Fig. 2A, B] and DG region [*F* (3, 15) = 7.189, *P* = 0.0032, Supplementary Fig. 2A, B]. In addition, western blotting analysis confirmed upregulated GSDMD and cleaved GSDMD with CLP, reversed by NSA but not by Mdivi-1 [*F* (3, 12) = 7.361, *P* = 0.0047; *F* (3, 12) = 10.41, *P* = 0.0012, Fig. 2C]. Moreover, immunostaining using an antibody against Drp1 revealed an increase in the mean intensity of Drp1 in the hippocampal CA1 region after CLP, but administration of NSA or Mdivi-1 rescued these changes [*F* (3, 15) = 6.400, *P* = 0.0052, Fig. 2D, E]. However, CLP did not significantly increase the mean intensity of Drp1 in hippocampal DG region [*F* (3, 15) = 1.845, *P* = 0.1824, Supplementary Fig. 2C, D]. Western blotting analysis verified that CLP upregulated Drp1 expression in the mouse hippocampus, while NSA or Mdivi-1 reversed this effect [*F* (3, 12) = 9.324, *P* = 0.0018, Fig. 2F].

To investigate a potential direct interaction between GSDMD and Drp1, Co-IP experiments were conducted using either an anti-GSDMD antibody or an anti-Drp1 antibody. Subsequently, the immunoprecipitates were tested with both anti-GSDMD and anti-Drp1 antibodies. Interestingly, a Drp1 band was clearly identified in the GSDMD immunoprecipitates, and conversely, a GSDMD band was detected in the Drp1 immunoprecipitates. These results suggest a direct interaction between GSDMD and Drp1 (Fig. 2G).

### Inhibiting GSDMD or Drp1 attenuated microglia and astrocytes activation and inflammatory cytokines release in the hippocampus of SAE mice

**Fig. 3:**
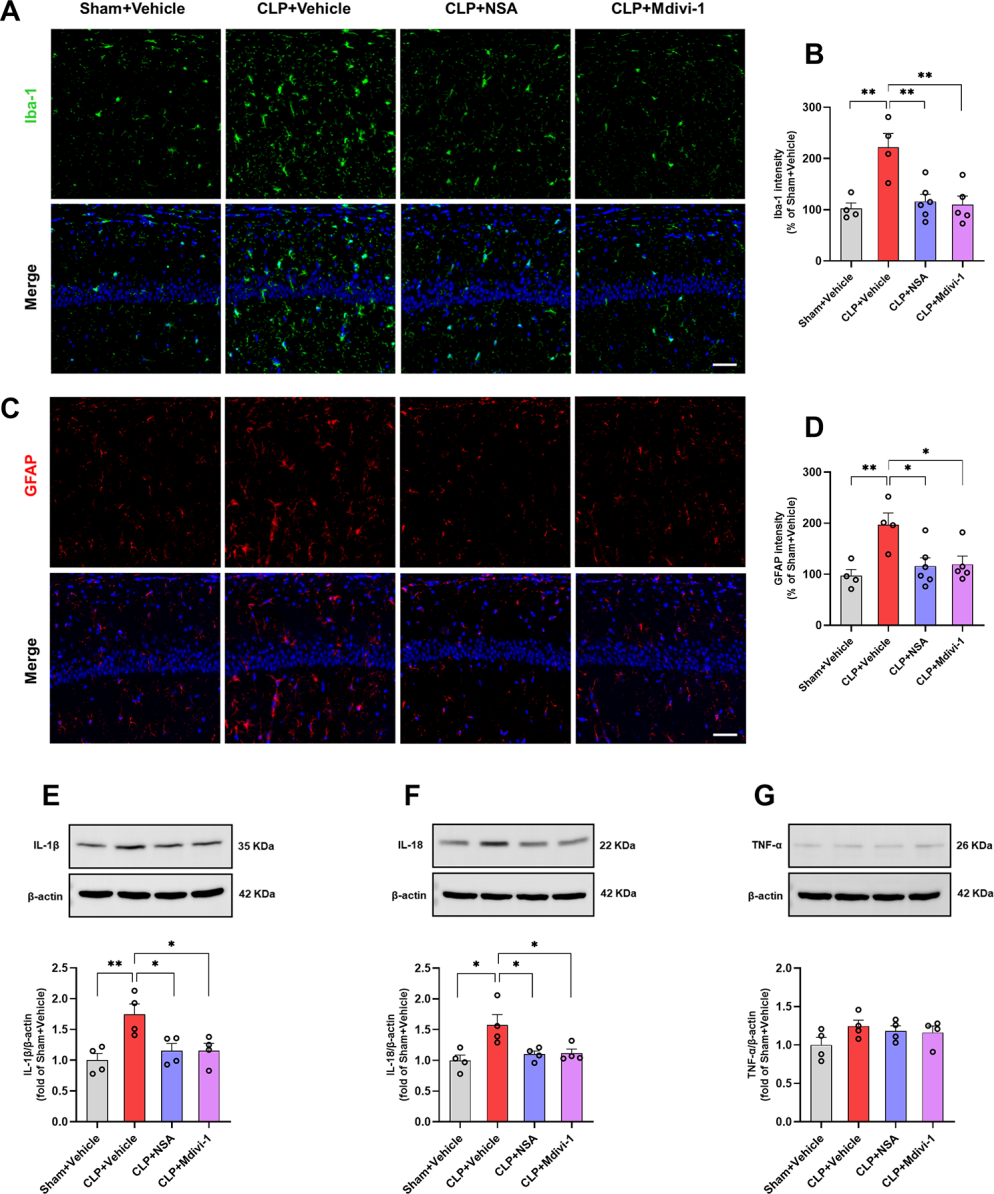
NSA or Mdivi-1 attenuated the activation of microglia and astrocytes and the release of inflammatory cytokines in the hippocampus of SAE mice. **A** Representative images of Iba-1 (green) in the hippocampal CA1 region. **B** Quantification of Iba-1 fluorescence hippocampal CA1 region. **C** Representative images of GFAP (red) in the hippocampal CA1 region. **D** Quantification of GFAP fluorescence hippocampal CA1 region. Data are presented as mean ± SEM (*n* = 4–6 mice/group). DAPI staining is shown in blue. Scale bar = 50 μm. **E**–**G** Representative western blotting and quantitative analysis of the protein levels of IL-1β, IL-18 and TNF-a in the hippocampus. Data are presented as the mean ± SEM (*n* = 4 mice/group). ^*^P < 0.05, ^**^P < 0.01 versus the indicated groups

Given the critical role of the hippocampus in memory and learning, especially its susceptibility to sepsis-induced neuroinflammation [10, 37], we performed morphological assessments of microglia and astrocytes in the hippocampus. Immunostaining with Iba-1 and GFAP antibodies revealed a significant increase in the mean intensity of Iba-1 [*F* (3, 15) = 8.999, *P* = 0.0012, Fig. 3A, B] and GFAP [*F* (3, 15) = 5.654, *P* = 0.0085, Fig. 3C, D] in the hippocampal CA1 region, as well as the DG region [Iba-1: *F* (3, 15) = 5.436, *P* = 0.0099, Supplementary Fig. 3A, B; GFAP: *F* (3, 15) = 6.669, *P* = 0.0044, Supplementary Fig. 3C, D] of the CLP + vehicle group, indicating microglial and astrocytic activation. Notably, treatment with NSA or Mdivi-1 effectively attenuated the activation of microglia and astrocytes in the hippocampus of mice with SAE.

Moreover, our findings indicated elevated levels of IL-1β [*F* (3, 12) = 6.335, *P* = 0.0080, Fig. 3E] and IL-18 [*F* (3, 12) = 5.927, *P* = 0.0101, Fig. 3F] in the hippocampus of CLP + vehicle mice, while NSA or Mdivi-1 treatment successfully reversed these increases. However, there was no significant change in TNF-α [*F* (3, 12) = 1.552, *P* = 0.2521, Fig. 3G] levels in the hippocampus following CLP challenge.

### Inhibiting GSDMD or Drp1 protected against mitochondrial and neuronal damage in the hippocampus of SAE mice

**Fig. 4:**
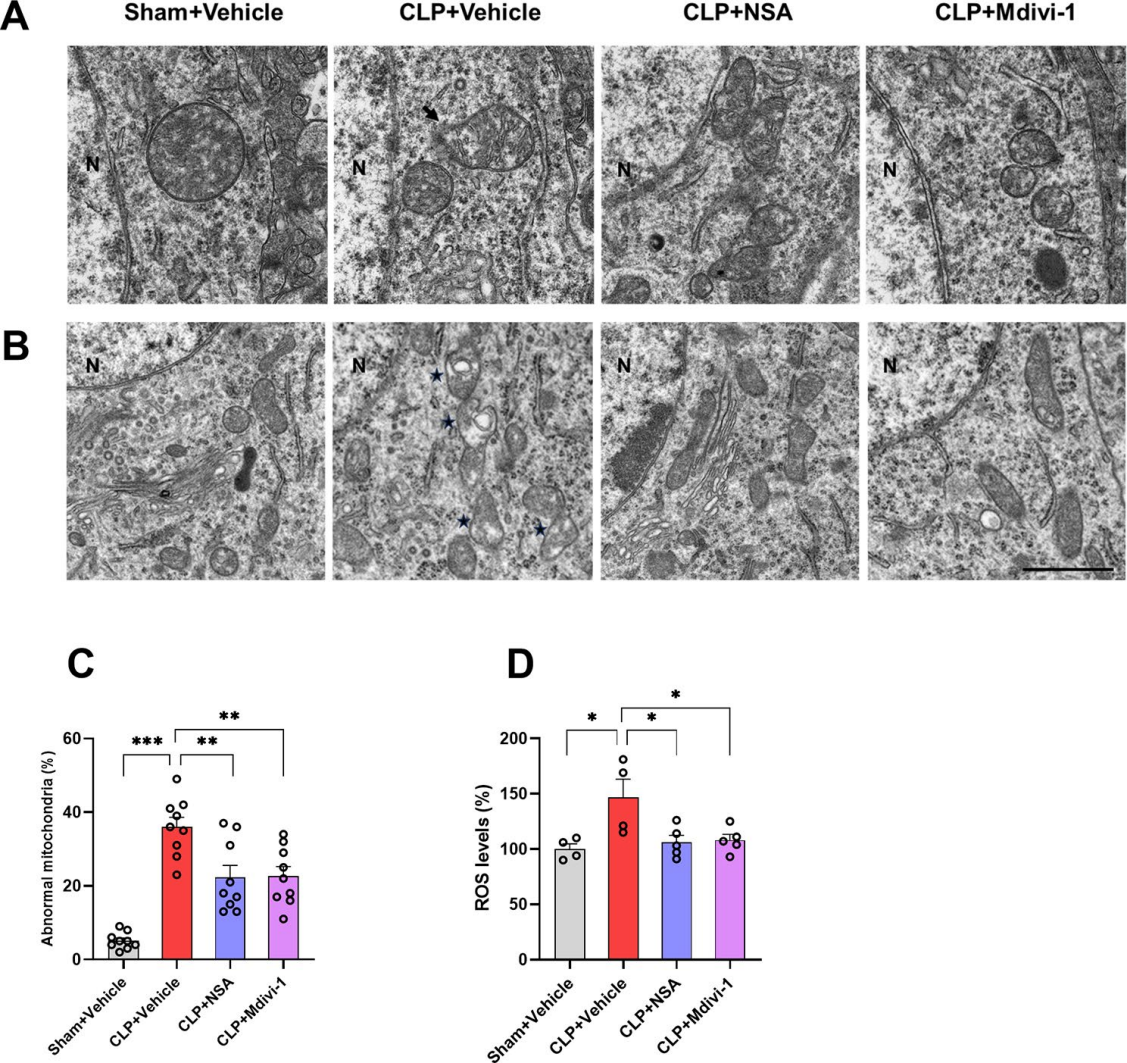
NSA or Mdivi-1 reversed mitochondrial abnormalities in the hippocampus of SAE mice. **A** Representative images of mitochondria ultrastructure surrounding the nuclei, the black arrow showed the interruptions of continuity of mitochondrial membrane. **B** Representative images of mitochondria ultrastructure in the hippocampal CA1 region. The asterisks showed the abnormal mitochondria. **C** Quantification of the abnormal mitochondria. Data are presented as the mean ± SEM (*n* = 3 mice/group, three images per mouse). Magnification 10,000 ×. Scale bar = 1 μm. **D** ROS levels in the hippocampus. Data are presented as the mean ± SEM (*n* = 4–5 mice/group). ^*^P < 0.05, ^**^P < 0.01, ^***^P < 0.001 versus the indicated groups

Mitochondrial dysfunction is associated with neuroinflammation and neurodegenerative disease [38, 39]. Recent evidence suggests that mitochondrial impairment contributes to proapoptotic processes, oxidative stress, and neuroinflammation [19]. This study explored the impact of NSA or Mdivi-1 on sepsis-induced mitochondrial damage by analyzing hippocampal mitochondria through TEM analysis. TEM revealed interruptions in the continuity of the mitochondrial membrane ultrastructure surrounding the nuclei in the CLP + vehicle group; however, which were ameliorated by NSA or Mdivi-1 treatment (Fig. 4A). In addition, compared with the sham + vehicle group, the number of abnormal mitochondria was increased remarkably in the CLP + vehicle group, which were rescued by NSA or Mdivi-1 treatment [*F* (3, 32) = 24.93, *P* < 0.0001, Fig. 4B, C]. To further evaluate CLP-induced mitochondrial damage and the protective effects of NSA or Mdivi-1, cellular ROS levels, indicative of mitochondrial damage, were measured [7, 40]. The results showed that sepsis-induced increases in ROS levels were repressed by the administration of NSA or Mdivi-1 [*F* (3, 14) = 5.199, *P* = 0.0127, Fig. 4D], indicating a restoration of mitochondrial functions.

**Fig. 5:**
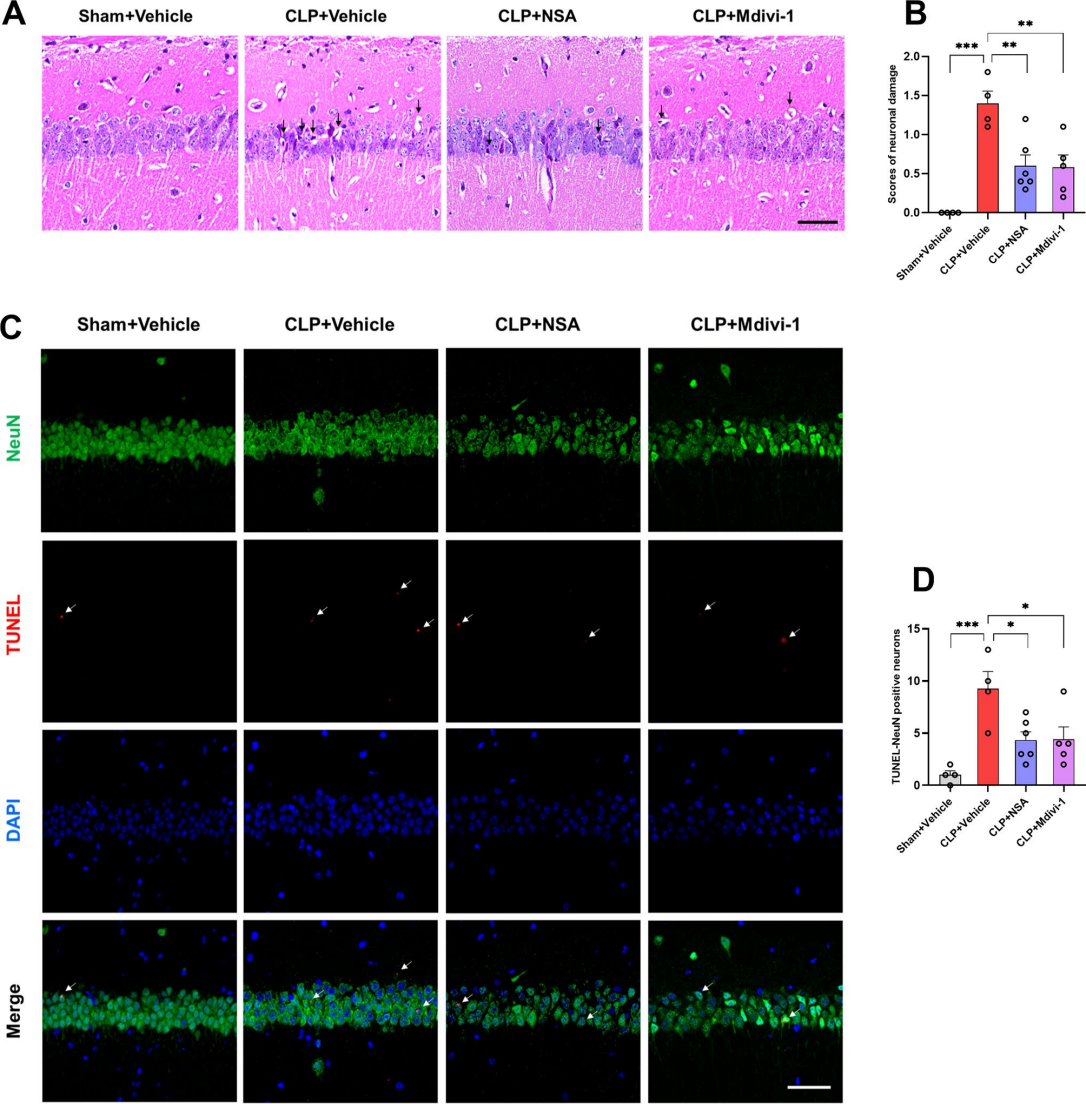
NSA or Mdivi-1 reduced neuronal damages in the hippocampus of SAE mice. **A** Representative drawing of HE staining showing morphologic appearance of neurons in the hippocampal CA1 region, the black arrows showed the marked nuclear pyknosis and necrotic neuron. Scale bar = 50 μm. **B** Semiquantitative analysis in the hippocampal neuronal damage scores. **C** Representative images of TUNEL staining (red) and NeuN (green) in the hippocampal CA1 region (the white arrows showed TUNEL-NeuN positive neurons). DAPI staining is shown in blue. Scale bar = 50 μm. **D** Numbers of TUNEL-NeuN positive neurons in the hippocampal CA1 region. Data are presented as the mean ± SEM (*n* = 4–6 mice/group). ^*^P < 0.05, ^**^P < 0.01, ^***^P < 0.001 versus the indicated groups

To evaluate the neuroprotective effects of NSA or Mdivi-1 on sepsis-induced neuronal damage, we performed HE and TUNEL staining on brain sections of hippocampal CA1 region of mice 16 days after surgery. HE staining of the hippocampus in the sham surgical group revealed intact neurons with large vesicular nuclei. In contrast, the CLP + vehicle group showed a marked increase in necrotic neurons characterized by shrunken neuronal bodies and nuclear pyknosis in the hippocampal CA1 region, resulting in elevated neuronal damage scores compared with the sham + vehicle group. Importantly, treatment with NSA or Mdivi-1 rescued hippocampal neuronal damage induced by CLP [*F* (3, 15) = 13.31, *P* = 0.0002, Fig. 5A, B].

TUNEL staining analysis further supported these findings, revealing a significantly higher number of TUNEL- NeuN positive neurons in the hippocampus CA1 of the CLP + vehicle group compared to the sham + vehicle group. In contrast, the CLP + NSA and CLP + Mdivi-1 groups demonstrated a decrease in TUNEL-NeuN positive neurons [*F* (3, 15) = 8.309, *P* = 0.0017, Fig. 5C, D]. Therefore, inhibition of GSDMD or Drp1 attenuated sepsis-induced neuronal damage in the hippocampus.

### Inhibiting GSDMD or Drp1 attenuated CLP-induced synaptic damage in the hippocampus of SAE mice

**Fig. 6:**
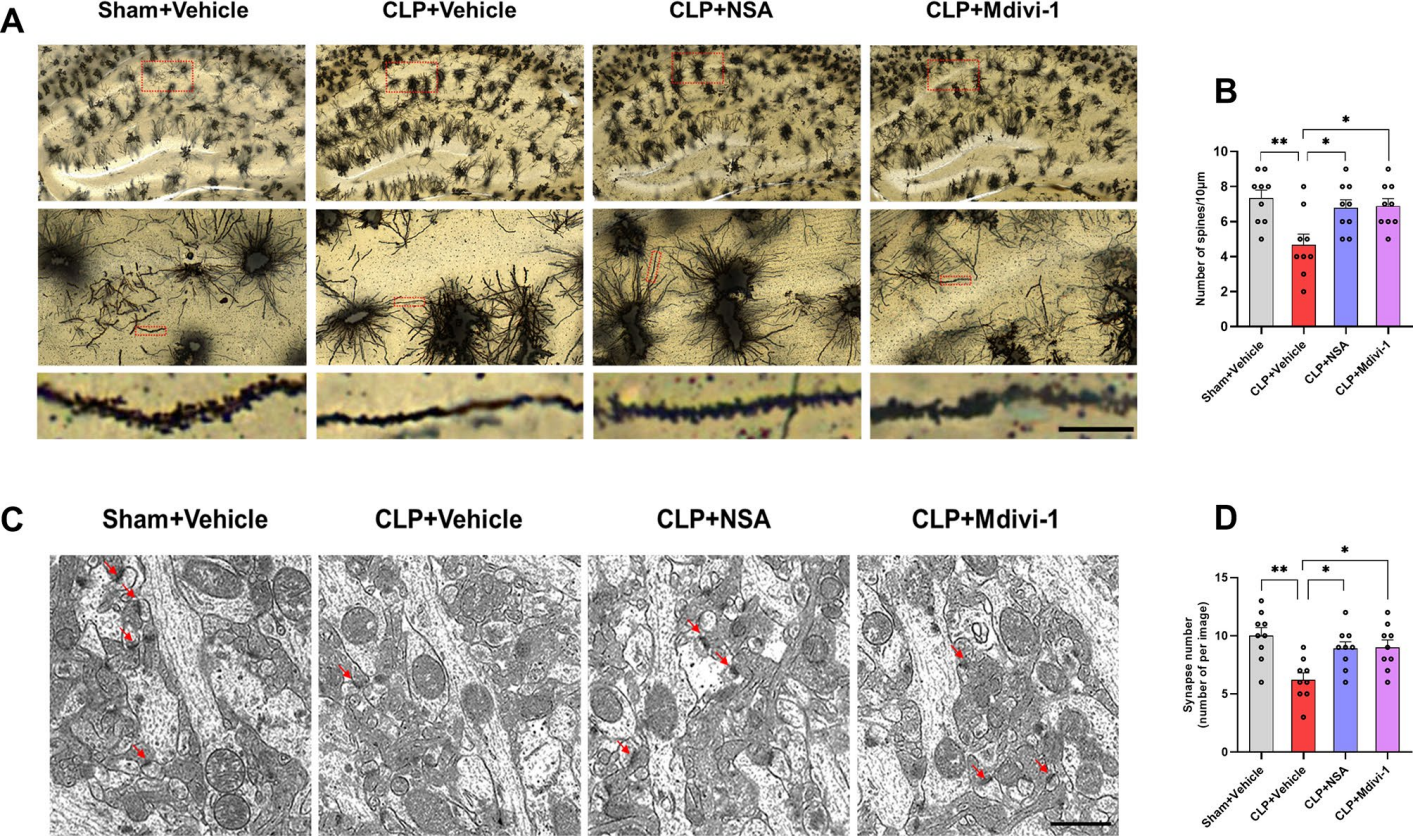
NSA or Mdivi-1 attenuated synaptic damages in the hippocampus of SAE mice. **A** Representative microphotograph of Golgi-Cox staining in the hippocampal sections. Magnification images from red box in upper panels. Scale bar = 10 μm. **B** Quantification of dendritic spine density of neurons in the hippocampus (*n* = 3 mice/group, three images per mouse). **C** Representative TEM images indicating the synapses in the longitudinal cross-section of an axon (indicated by red arrows). Magnification 3,000 ×. Scale bar = 2 μm. **D** Synaptic counts in the hippocampus. Data are presented as the mean ± SEM (*n* = 3 mice/group, three images per mouse). ^*^P < 0.05, ^**^P < 0.01 versus the indicated groups

The connection between cognitive impairment and synaptic abnormalities in the hippocampus during SAE remains unclear. Previous evidence suggests that Drp1 is integral to synapse formation [31]. To evaluate whether inhibiting GSDMD or Drp1 could attenuate synaptic damage in the hippocampus of septic mice, we used Golgi staining to analyze the morphology and assess the spine density of neurons in the hippocampus. As shown in Fig. 6, compared with the sham + vehicle group, the CLP + vehicle group showed a significantly lower dendritic spine density in the hippocampal CA1 region. However, treatment with NSA or Mdivi-1 attenuated the CLP-induced loss of dendritic spines [*F* (3, 32) = 5.341, *P* = 0.0043, Fig. 6A, B]. In addition, we observed a notable reduction in the number of synapses in the hippocampal CA1 region of CLP + vehicle mice compared to the sham + vehicle group mice. However, treatment with NSA or Mdivi-1 attenuated the sepsis-induced decrease in the number of synapses in the hippocampal CA1 region [*F* (3, 32) = 6.461, *P* = 0.0015, Fig. 6C, D].

### Inhibiting GSDMD or Drp1 reversed the decrease in neural oscillations in the hippocampus of SAE mice

**Fig. 7:**
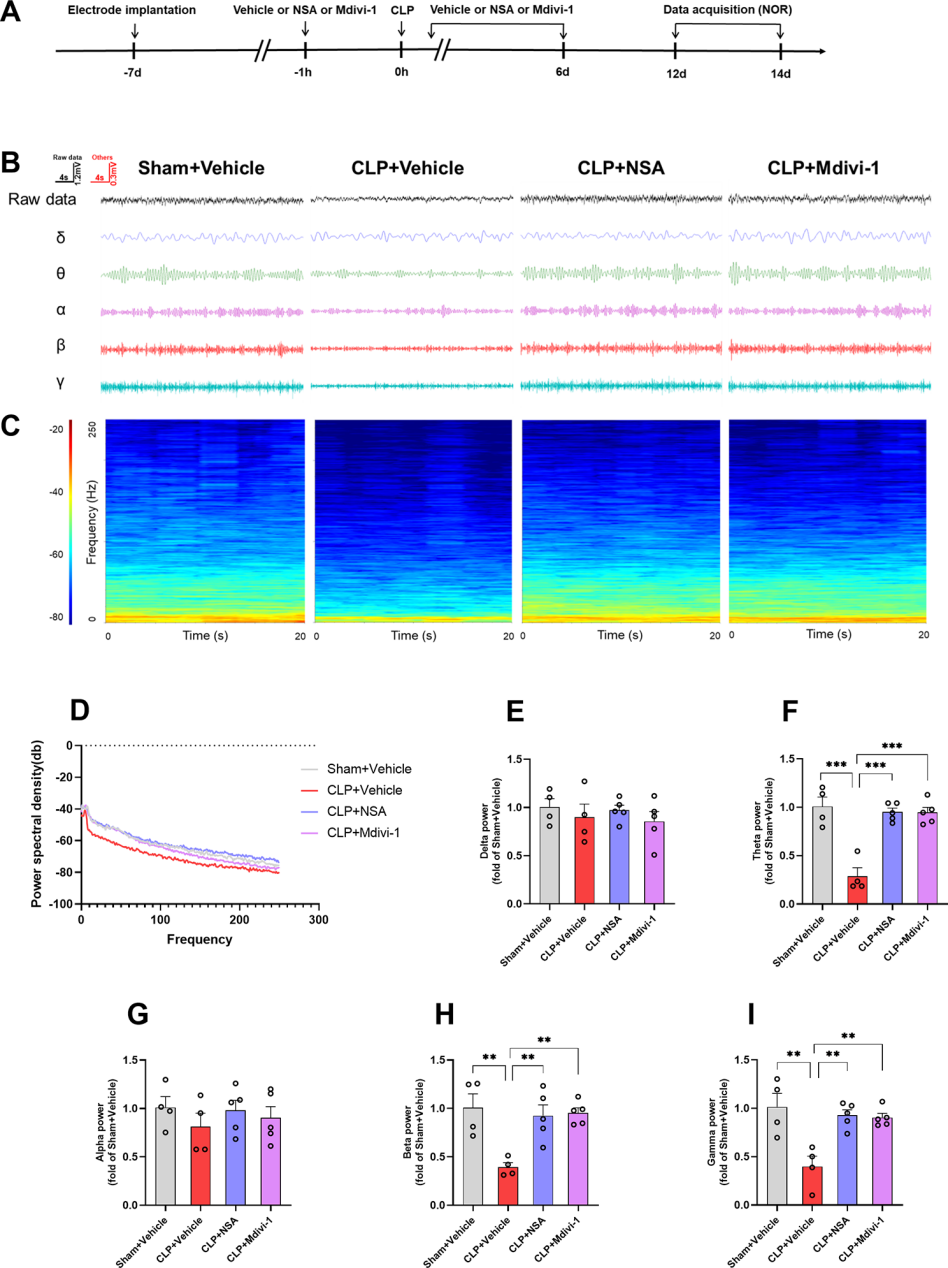
NSA or Mdivi-1 reversed the decrease in theta, beta, and gamma band power in the hippocampus of SAE mice. **A** Schematic timeline of the electrophysiological experiment. **B** Representative images of local field potential and filtered delta, theta, alpha, beta, and gamma band in the hippocampus. **C** Time frequency diagrams in the hippocampus. **D** Example power spectra of local field potential in the hippocampus. **E**–**I** Quantification of average delta, theta, alpha, beta, and gamma band power in the hippocampus. Data are presented as the mean ± SEM (*n* = 4–5 mice/group). ^**^P < 0.01, ^***^P < 0.001 versus the indicated groups

To further evaluate the role of NSA and Mdivi-1 in altered oscillatory activities associated with CLP-induced cognitive impairments, we measured LFP in the hippocampal CA1 region during the NOR test. As shown in Fig. 7, in the present study, compared to the sham + vehicle group, the power of theta, beta, and gamma oscillations significantly decreased in the CLP + vehicle group. Importantly, these deficits were effectively reversed by treatment with NSA or Mdivi-1 [delta: *F* (3, 14) = 0.4976, *P* = 0.6899; theta: *F* (3, 14) = 22.49, *P* < 0.0001; alpha: *F* (3, 14) = 0.5261, *P* = 0.6715; beta: *F* (3, 14) = 8.132, *P* = 0.0022; gamma: *F* (3, 14) = 0.9217, *P* = 0.0013).

## Discussion

**Fig. 8:**
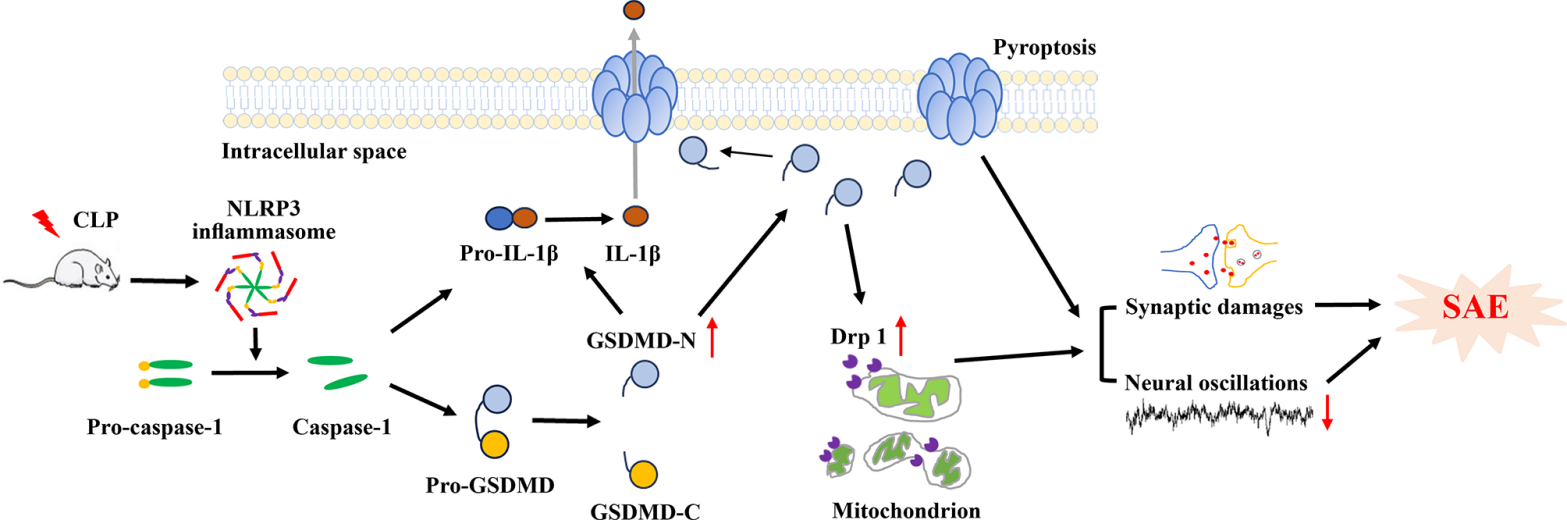
The schematic diagram illustrates that CLP induces the activation of GSDMD and subsequently increases Drp1, leading to mitochondrial injury, significant neuronal and synaptic damage, and a decrease in neural oscillations in the hippocampus, ultimately contributing to cognitive impairments. Notably, inhibiting GSDMD/Drp1 pathway might reverse these aberrant changes and ameliorate cognitive deficits in SAE mice

This study reveals, for the first time, that cognitive impairment induced by sepsis involves the activation of GSDMD, increased levels of Drp1, mitochondrial injury, resulting in synaptic damage, and disrupted neural oscillations in the hippocampus. Remarkably, inhibiting the GSDMD/Drp1 pathway shows promise in reversing these pathological changes and ameliorating cognitive deficits in a mouse model of SAE (Fig. 8).

Our results suggested that sepsis induced by CLP leads to cognitive impairments, consistent with prior research [30]. Notably, treatment with the GSDMD-specific inhibitor NSA or Drp1-specific inhibitor Mdivi-1 rescued deficits in discrimination ratio, spontaneous alteration, and improved hippocampus-dependent memory in SAE mice. The hippocampus, with its high expression of cytokine receptors, is susceptible to the detrimental effects of proinflammatory cytokines, as demonstrated in our previous research revealing NLRP3-mediated hippocampal neuroinflammation and pyroptosis leading to learning and memory deficits in septic mice [10].

GSDMD, an executor of various cell death pathways, can lyse liposomes and form pores on the cell membrane, releasing the proinflammatory cytokine IL-1β [13, 20]. The present study confirms that GSDMD is indeed involved in the release of proinflammatory cytokines IL-1β and IL-18 in the context of SAE, consistent with previous research [10]. Sepsis-induced nuclear autophagy might be linked to mitochondrial damage through GSDMD, providing a potential mechanistic insight into the pathology of SAE [12]. The study further explores the therapeutic potential of targeting GSDMD-mediated mitochondrial damage in the treatment of SAE. Notably, NSA treatment mitigated these abnormalities induced by CLP in the hippocampus of SAE mice. However, Mdivi-1 treatment did not attenuate the activation of GSDMD after CLP, suggesting that Drp1 acts as a downstream factor of GSDMD in SAE.

Mitochondrial dysfunction plays a crucial role in neurocognitive diseases, contributing to apoptotic pathways and inflammation [7, 41]. Drp1 regulates mitochondrial dynamics and is implicated in mitochondrial aberrations, synaptic loss, and neuroinflammation. Pharmacological inhibition of Drp1 with Mdivi-1 has shown promise in attenuating cognitive deficits and synaptic dysfunctions in conditions like intracerebral hemorrhage [42]. It also shows promise in alleviating neuroinflammation and neuron apoptosis by inhibiting microglia and astrocyte activation in subarachnoid hemorrhage [43]. Recent studies indicate that Drp1 is upregulated in the brain tissue of septic mice, suggesting Mdivi-1 as a potential therapeutic approach for sepsis-related brain damage [44, 45]. In the animal model of CLP-induced SAE, our data reveal significant Drp1 activation, leading to mitochondrial, neuronal, and synaptic damage in the hippocampus. Notably, Mdivi-1 effectively reverses these changes by inhibiting Drp1 upregulation.

Our previous study highlighted the potential of the mitochondrion-targeted peptide SS-31 in preventing mitochondrial dysfunction, inhibiting neuroinflammation, and alleviating cognitive deficits in SAE mice [7]. The ability of SS-31 to inhibit activation of the NLRP3 inflammasome, cleavage of GSDMD, and reduce the mitochondrial recruitment of GSDMD-N [46] further supports the idea that protecting mitochondria could be a potential target for GSDMD-mediated disorders. Our data reinforce the notion that, in addition to GSDMD’s involvement in the release of proinflammatory cytokines, activated GSDMD may participate in the Drp1 signaling pathway. We unequivocally demonstrate that activated GSDMD acts as an upstream factor, causing Drp1 activation, neuronal damage, synaptic abnormalities, and altered neural oscillations in SAE mice. This cascade of events is effectively reversed by treatment with NSA or Mdivi-1, suggesting that activated GSDMD may promote the translocation of Drp1 to the mitochondrial membrane, subsequently provoking mitochondrial dysfunctions [11, 47, 48].

Mitochondria serve as an essential node for supplying energy and supporting neural oscillatory activities, which are essential for cognitive processes [49]. Altered oscillatory rhythmic activity is associated with cognitive impairments, as observed in conditions like AD [50]. Our results demonstrate that CLP-induced Drp1 upregulation and mitochondrial damage lead to abnormal neural oscillations, impacting memory performance, consistent with previous studies [30]. Importantly, inhibiting the GSDMD/Drp1 pathway effectively reverses neuronal injury, synaptic damage, and abnormalities in neural oscillations in the hippocampus of septic mice, thereby improving cognitive deficits.

This present study has a few limitations. Firstly, the administration of NSA or Mdivi-1 was limited to seven days, and the potential long-term adverse effects of NSA or Mdivi-1 treatment need thorough evaluation. Secondly, although the hippocampus was identified as a crucial contributor to CLP-induced cognitive impairments, future investigations should explore the involvement of other brain regions in the SAE process. Thirdly, to comprehensively assess mitochondrial dysfunction, additional parameters such as mitochondrial membrane potential, ATP levels, and ROS levels need to be measured. Lastly, optogenetic or chemogenetic techniques were not employed in this study and should be used in future research.

## Conclusions

Taken together, our findings suggest that intervention in the GSDMD/Drp1 pathway, specifically using NSA or Mdivi-1, may partially alleviate neuroinflammation, mitigate mitochondrial changes, attenuate neuronal and synaptic damage, rectify abnormalities in neural oscillations, and ultimately ameliorate cognitive deficits in a murine model of SAE.

### Electronic supplementary material

Below is the link to the electronic supplementary material.

**Supplementary Material 1: Supplementary Fig. 1** NSA or Mdivi-1 did not influence the behavioral analysis of mice after sham surgery. **A** Total ambulatory distance in open field tests. **B** Time spent in the center in open field tests. **C** Discrimination ratio in the novel object recognition tests. **D** Spontaneous alterations in Y maze tests. Data are presented as the mean ± SEM (*n* = 10 mice/group)

**Supplementary Material 2: Supplementary Fig. 2** Effect of NSA or Mdivi-1 treatment on GSDMD and Drp1 fluorescence in the hippocampal DG region. **A** Representative images of GSDMD (green) in the hippocampal DG region. **B** Quantification of GSDMD fluorescence. **C** Representative images of Drp1 (red) in the hippocampal DG region. **D** Quantification of Drp1 fluorescence. Data are presented as the mean ± SEM (*n* = 4–6 mice/group). ^*^P < 0.05, ^**^P < 0.01 versus the indicated groups. DAPI staining is shown in blue. Scale bar = 50 μm

**Supplementary Material 3: Supplementary Fig. 3** NSA or Mdivi-1 attenuated activation of microglia and astrocytes in the hippocampal DG region. **A** Representative images of Iba-1 (green) in the hippocampal DG region. **B** Quantification of Iba-1 fluorescence. **C** Representative images of GFAP (red) in the hippocampal DG region. **D** Quantification of GFAP fluorescence. Data are presented as mean ± SEM (*n* = 4–6 mice/group). ^*^P < 0.05, ^**^P < 0.01 versus the indicated groups. DAPI staining is shown in blue. Scale bar = 50 μm

**Supplementary Material 4**

## Data Availability

All data generated or analyzed during this study are included in this published article or its supplementary information files.

## Abbreviations

SAE: Sepsis‑associated encephalopathy
POCD: Postoperative cognitive dysfunction
AD: Alzheimer’s disease
PD: Parkinson’s disease
GSDMD: Gasdermin D
Drp1: Dynamin-related protein 1
CLP: Cecal ligation and puncture
OF: Open field
NOR: Novel object recognition
i.p.: Intraperitoneally
BBB: Blood-brain barrier
NSA: Necrosulfonamide
Mdivi-1: Mitochondrial division inhibitor-1
Iba-1: Ionized calcium-binding adaptor molecule-1
GFAP: Glial fibrillary acidic protein
NLRP3: Nucleotide-binding domain-like receptor protein 3
IL-1β: Interleukin-1β
Aβ: Amyloid-β
Lrrk2: Leucine-rich repeat kinase 2
DAPI: 4′,6-diamidino-2-phenylindole
HE: Hematoxylin and eosin
TEM: Transmission electron microscopy
LFP: Local field potential
