# Supplementary figures and images for: GSDMD/Drp1 signaling pathway mediates hippocampal synaptic damage and neural oscillation abnormalities in a mouse model of sepsis-associated encephalopathy
Source: J Neuroinflammation. 2024 Apr 16;21:96. doi: 10.1186/s12974-024-03084-w (PMC11020266; doi:10.1186/s12974-024-03084-w)

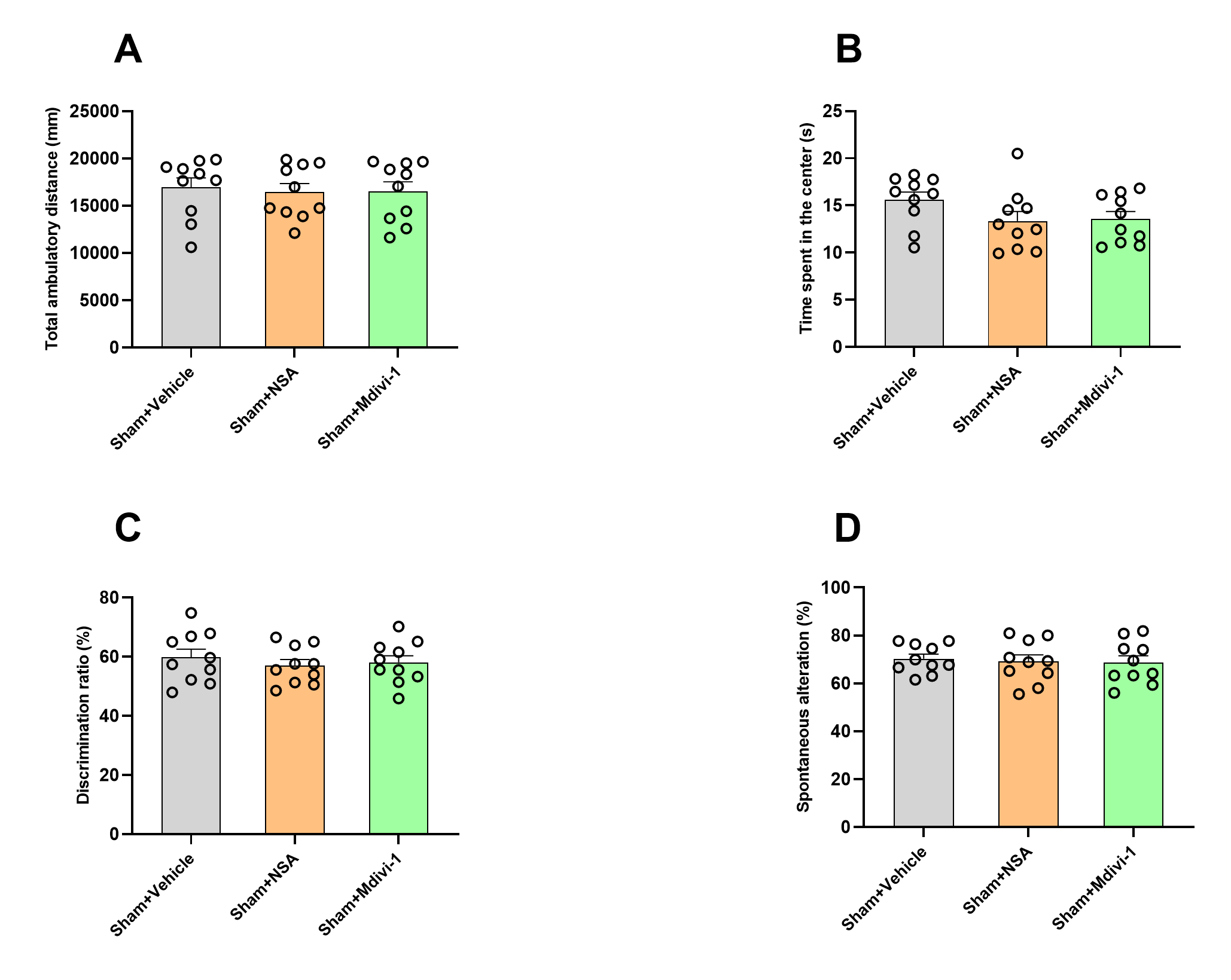

Supplement: Supplementary file 1 — Supplementary Material 1: Supplementary Fig. 1 NSA or Mdivi-1 did not influence the behavioral analysis of mice after sham surgery. A Total ambulatory distance in open field tests. B Time spent in the center in open field tests. C Discrimination ratio in the novel object recognition tests. D Spontaneous alterations in Y maze tests. Data are presented as the mean ± SEM (n = 10 mice/group) [file 12974_2024_3084_MOESM1_ESM.tif]

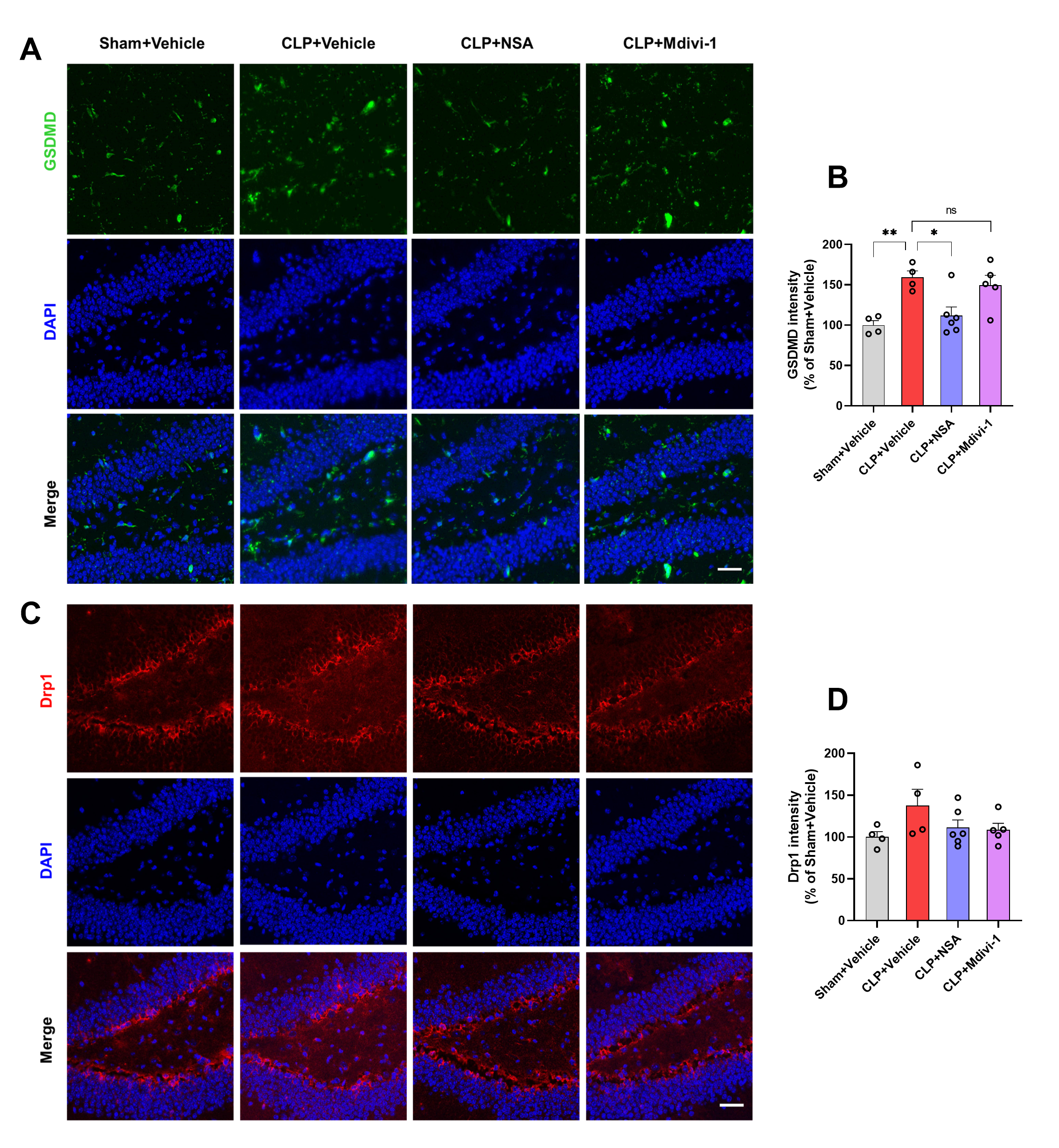

Supplement: Supplementary file 2 — Supplementary Material 2: Supplementary Fig. 2 Effect of NSA or Mdivi-1 treatment on GSDMD and Drp1 fluorescence in the hippocampal DG region. A Representative images of GSDMD (green) in the hippocampal DG region. B Quantification of GSDMD fluorescence. C Representative images of Drp1 (red) in the hippocampal DG region. D Quantification of Drp1 fluorescence. Data are presented as the mean ± SEM (n = 4–6 mice/group). *P < 0.05, **P < 0.01 versus the indicated groups. DAPI staining is shown in blue. Scale bar = 50 μm [file 12974_2024_3084_MOESM2_ESM.tif]

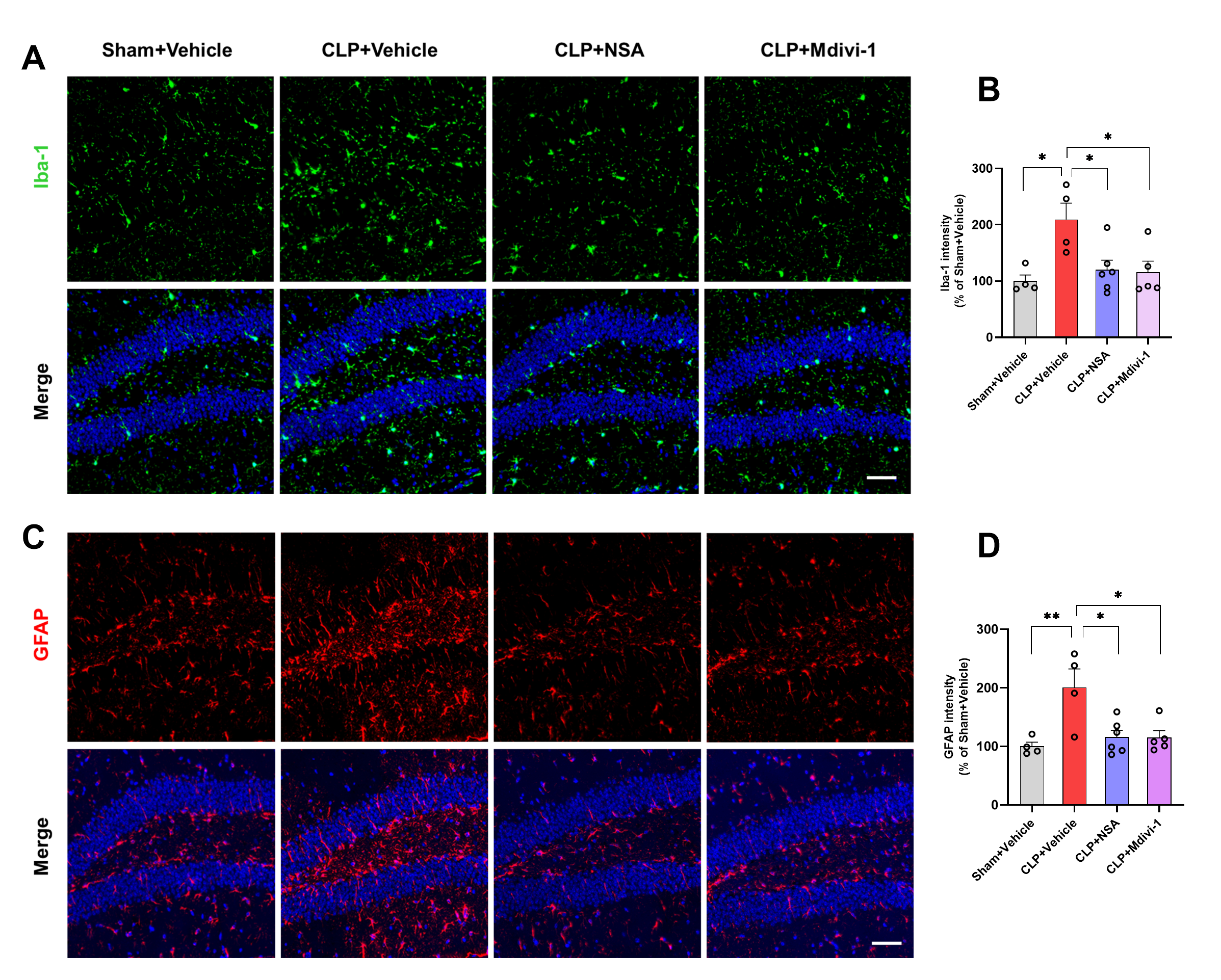

Supplement: Supplementary file 3 — Supplementary Material 3: Supplementary Fig. 3 NSA or Mdivi-1 attenuated activation of microglia and astrocytes in the hippocampal DG region. A Representative images of Iba-1 (green) in the hippocampal DG region. B Quantification of Iba-1 fluorescence. C Representative images of GFAP (red) in the hippocampal DG region. D Quantification of GFAP fluorescence. Data are presented as mean ± SEM (n = 4–6 mice/group). *P < 0.05, **P < 0.01 versus the indicated groups. DAPI staining is shown in blue. Scale bar = 50 μm [file 12974_2024_3084_MOESM3_ESM.tif]
